# Circulating Nesfatin-1 Levels and Type 2 Diabetes: A Systematic Review and Meta-Analysis

**DOI:** 10.1155/2017/7687098

**Published:** 2017-12-28

**Authors:** Ting Zhai, Shi-Zhen Li, Xin-Tong Fan, Zhao Tian, Xiao-Qing Lu, Jing Dong

**Affiliations:** ^1^Preventive Medicine Department, Grade 2014, School of Public Health, Qingdao University, Shandong, China; ^2^Clinical Medicine Department, Grade 2014, Medical College, Qingdao University, Shandong, China; ^3^School of Public Health, Qingdao University, Qingdao, China; ^4^Physiology Department, Medical College, Qingdao University, Shandong, China; ^5^Special Medicine Department, Medical College, Qingdao University, Shandong, China

## Abstract

The role of nesfatin-1 in glucose homeostasis has been investigated previously. However, although numerous studies have examined the relationships between circulating nesfatin-1 levels and type 2 diabetes, the conclusions are contradictory. We aimed to probe the relationship between circulating nesfatin-1 levels and type 2 diabetes by meta-analysis. Seven studies including 328 type 2 diabetes patients and 294 control subjects were included. Although there was no obvious difference in circulating nesfatin-1 levels between patients with type 2 diabetes and the control group (MD = −0.04; 95% CI = −0.32 to −0.23), subgroup analysis showed higher nesfatin-1 levels in newly diagnosed type 2 diabetes patients (MD = 0.59; 95% CI = 0.45 to 0.74) and significantly lower nesfatin-1 levels in type 2 diabetes patients receiving antidiabetic treatment (MD = −0.26; 95% CI = −0.33 to −0.20). In conclusion, the analysis supports a relationship between circulating nesfatin-1 levels and type 2 diabetes, where newly diagnosed type 2 diabetes was associated with an elevated Nesfatin-1 level, and type 2 diabetes patients receiving antidiabetic treatment showed lower circulating nesfatin-1 levels.

## 1. Introduction

Nesfatin-1 was first identified as an anorexigenic neuropeptide originating from nucleobindin-2 (NUCB2) [1]. Central injection of nesfatin-1 inhibited dark-phase food intake and was accompanied by a chronic reduction in body weight gain and fat pads. Recent studies also revealed an antihyperglycemic role of nesfatin-1 in glucose homeostasis [2]. Data has shown that nesfatin-1 may act in the brain to regulate insulin sensitivity [3]. Additionally, an effect of nesfatin-1 to increase insulin release in beta cells under hyperglycemic conditions has been described [4], and nesfatin-1 can cross the brain-blood barrier bidirectionally in a nonsaturable manner [5]. These pieces of evidence suggest an important role of circulating nesfatin-1 in energy homeostasis.

Currently, there are few sufficient therapeutic options for patients with type 2 diabetes and new insights into the pathogenesis of this disease are urgently needed. Since the levels of nesfatin-1 affect energy homeostasis, type 2 diabetes may also be affected [6, 7]. However, studies evaluating the relationship between circulating nesfatin-1 and type 2 diabetes have produced conflicting results. Some studies revealed high levels of nesfatin-1 in patients with type 2 diabetes [8, 9], but others reported lower nesfatin-1 levels in these patients [10–14]. Additionally, the potential causes of these conflicting results have been poorly described.

In this meta-analysis, we aimed to clarify the association between circulating nesfatin-1 levels and type 2 diabetes.

## 2. Methods

We observed the Meta-Analyses and Systematic Reviews of Observational Studies (MOOSE) guidelines for this analysis [15]. The included studies are searched according to the Preferred Reporting Items for Systematic Reviews and Meta-Analyses (PRISMA) statement [16].

### 2.1. Data Sources and Searches

A systematic literature search was carried out using four online databases, Embase, PubMed, the Cochrane Library, and the Web of Science up to January 2017. The key terms included in the search strategy are *nesfatin-1*, *nesfatin*, *NUCB2, nucleobindin*, *CALNUC*, *diabetes*, *diabetes mellitus*, *diabetes mellitus*, *type 2*, *type 2 diabetes*, *prediabetic*, *diabetic*, *glycemic*, *glycaemia*, *glucose tolerance*, and *insulin sensitivity.* The searching method was performed without language restrictions. When there was ambiguity about the results or insufficient data for analysis, we contacted the authors to attempt to obtain the necessary data. We also browsed the references of included papers for potentially relevant publications.

### 2.2. Study Selection

We considered studies eligible if they met the following inclusion criteria: (1) Patients with type 2 diabetes were used as the case group, and the control group consisted of healthy people with normal glucose tolerance (NGT); (2) all type 2 diabetes patients had no other complications; and (3) reported circulating nesfatin-1 levels were described sufficiently for calculation. Studies were excluded based on the following exclusion criteria: (1) Study information available only as abstracts, reviews, case reports, editor opinions, or expert comments; (2) animal or in vitro experimentation; and (3) lacking necessary data required for analyses.

### 2.3. Data Extraction and Quality Assessment

Two independent reviewers (Zhai and Li) browsed all included studies and extracted data from each study into a predefined spreadsheet. If there was a discrepancy, the reviewers assessed the data together to come to an agreement. The gathered information included name of the first author, publication year, country, study design, method of measuring circulating nesfatin-1, type 2 diabetes diagnosis criteria, acceptance of antidiabetic treatment, duration of type 2 diabetes, size of case and control groups, circulating nesfatin-1 levels (presented as the mean with standard deviation), age, gender, body mass index (BMI), and other baseline parameters.

We used the Newcastle-Ottawa Scale (NOS) [17] to evaluate the quality of the included case-control studies (Supplementary Table 2A). The quality assessment criteria were as follows: (1) Whether the definition of type 2 diabetes was adequate with independent substantiation; (2) whether the type 2 diabetes cases were typical; (3) whether the control group subjects were from the same community; (4) whether the controls were described as having no preexisting type 2 diabetes or other type 2 diabetes-related diseases; (5) whether the case and control groups were matched or adjusted for age or BMI; (6) whether at least one additional element, such as waist-hip ratio or diet type, was matched for case and control groups; (7) whether the study of exposure was performed under blind measurement; (8) whether the cases and controls were assessed with the same test methods; and (9) whether the cases and controls exhibited the same nonresponse rates. Each criterion was scored as 0 or 1 based on how well the criterion was met. Studies with scores equal to or greater than seven were classified as high-quality studies, and other studies were classified as moderate quality studies. The maximum score attainable was 9.

We used the checklist recommended by the Agency for Healthcare Research and Quality (AHRQ) [18] to evaluate the quality of the included cross-sectional studies (Supplementary Table 2B). The quality assessment criteria were as follows: (1) Define the source of information (survey and record review); (2) list inclusion and exclusion criteria for exposed and unexposed subjects (cases and controls) or refer to previous publications; (3) indicate time period used for identifying patients; (4) indicate whether or not subjects were consecutive if not population-based; (5) indicate if evaluators of subjective components of the study were masked to other aspects of the status of the participants; (6) describe any assessments undertaken for quality assurance purposes (e.g., test/retest of primary outcome measurements); (7) explain any patient exclusions from analysis; (8) describe how confounding was assessed and/or controlled; (9) if applicable, explain how missing data were handled in the analysis; (10) summarize patient response rates and completeness of data collection; (11) clarify what follow-up, if any, was expected and the percentage of patients for which incomplete data or follow-up was obtained. The maximum score attainable was 11, and studies with scores equal to or greater than eight were classified as high-quality studies.

### 2.4. Data Synthesis and Analysis

We calculated the mean difference (MD) with 95% confidence intervals (CIs) for circulating levels in type 2 diabetes groups versus controls. Cochran's Q (chi-square) test was used to verify and *I* ^2^ was used to evaluate the heterogeneity among these studies. *I* ^2^> 50% suggests considerable heterogeneity [19]. We used a random effect model to pool the estimates [20]. Subgroup analysis according to antidiabetic treatment, average age, BMI, HOMA-IR ratio, blood samples, diagnosis criteria, study type, and study quality was performed and used to detect potential heterogeneity. Potential publication bias was evaluated by the Egger's test [21] and Begg's test [22]. For most tests, *P* value < 0.05 indicated a significant statistical difference. For the Cochran's Q test for heterogeneity, a significant value was considered 0.1. All data were analyzed with the Review Manager (RevMan 5.3) statistical software and Stata 12.0.

## 3. Results

### 3.1. Literature Search

**Figure 1:**
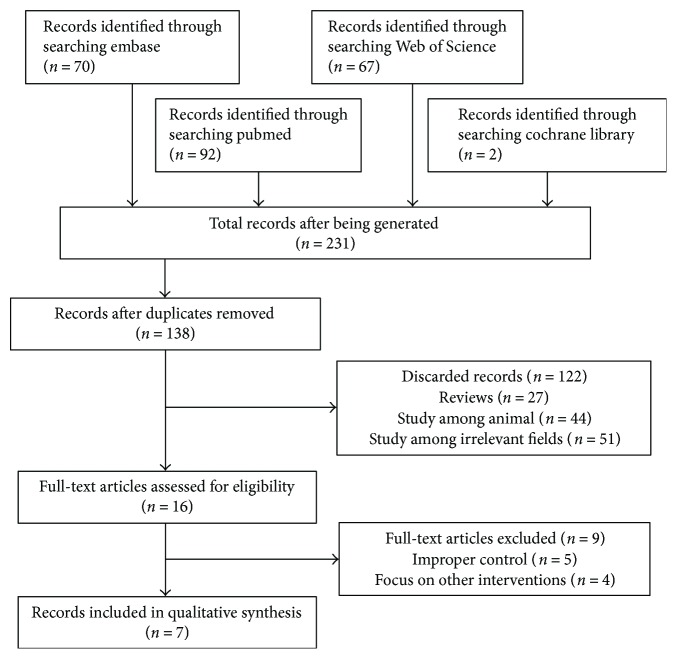
Flow chart for selection of eligible studies.

The detailed steps of the literature search are presented in Figure 1. The search strategy allowed identification of 231 reports from the four databases. After duplicates were removed, the titles and abstracts of 138 records were independently screened by two reviewers, resulting in the selection of 16 articles that were then assessed for eligibility. Finally, 7 reports were selected for inclusion in this meta-analysis [8–14].

### 3.2. Study Characteristics

The seven selected studies were published from 2010 to 2017 and together included 328 patients with type 2 diabetes and 294 healthy controls. The characteristics of the studies are presented in Table 1 and Supplemental Table 1. Six studies were carried out in China [9–14] and one in Turkey [8]. Six studies were in English [8–13] and one was Chinese [14]. Three studies were cross-sectional studies [9–11] and four were case-control studies [8, 12–14]. The diagnosis of type 2 diabetes was based on the criteria from the American Diabetes Association (ADA) in three studies [10–12] and the World Health Organization (WHO) in four studies [8, 9, 13, 14]. Two studies recruited patients with newly diagnosed type 2 diabetes [8, 9], and patients in the other five studies were type 2 diabetes patients who had already received antidiabetic treatment [10–14]. Only Li et al. described the specific antidiabetic treatments, either with insulin together with oral hypoglycemic agents or oral hypoglycemic agents only [13]. Only three studies reported the duration of type 2 diabetes [15–17]. Circulating nesfatin-1 levels in all included studies were examined after overnight fasting. Blood samples were obtained from the serum in three studies [10, 11, 14] and from plasma in four studies [8, 9, 12, 13] and were all measured by enzyme-linked immunosorbent assay (ELISA). The group size ranged from 20 to 74 in the included studies.

The quality assessment of the included studies is presented in Supplementary Table 2.

### 3.3. Overall Analysis

**Figure 2:**
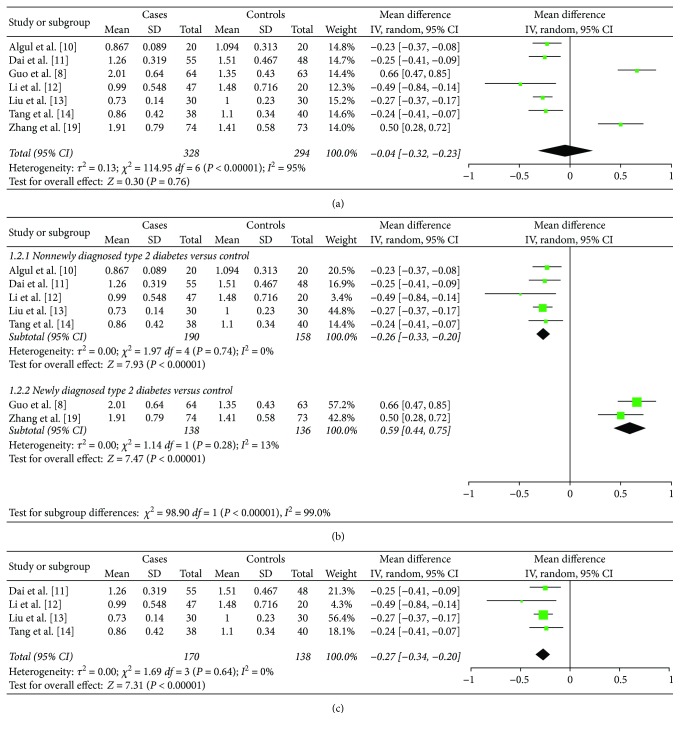
Forest plot of circulating nesfatin-1 levels and type 2 diabetes. (a) Overall meta-analysis of circulating nesfatin-1 levels in type 2 diabetes (random effects model). (b) Subgroup analysis of nesfatin-1 in newly diagnosed type 2 diabetes or antidiabetic treatment (random effects model). (c) Subgroup analysis of type 2 diabetes with treatment from China (random effects model).

As illustrated in Figure 2(a), there were no obvious differences in the circulating nesfatin-1 levels between the type 2 diabetes group and control group [MD = −0.04; 95% CI (−0.32 and −0.23), *P* = 0.76]. The MD from studies that exhibited significant heterogeneity was assessed by a random effect model (*I* ^2^ = 95%, *P* < 0.00001). Publication bias was evaluated and was considered insignificant (Begg's test: *P* = 0.368; Egger' test: *P* = 0.375).

### 3.4. Subgroup Analysis

Subgroup analysis was carried out to explore the source of the heterogeneity. We found that low or high nesfatin-1 level was related to whether patients received treatment for type 2 diabetes. Therefore, we divided these studies into two subgroups: the newly diagnosed type 2 diabetes subgroup (patients not receiving treatment) and the antidiabetic treatment subgroup, and the results are shown in Figure 2(b). Five studies were included in the antidiabetic treatment subgroup [10–14], and analysis revealed that circulating nesfatin-1 levels were significantly lower in patients with antidiabetic treatment than the levels in controls [MD = −0.26; 95% CI (−0.33 and −0.20), *P* < 0.00001, *I* ^2^ = 0%]. Two studies were included in the newly diagnosed type 2 diabetes subgroup [8, 9], and these patients showed increased nesfatin-1 levels compared with control subjects [MD = 0.59; 95% CI (0.45 and 0.74), *P* < 0.00001, *I* ^2^ = 13%]. Although *I* square of both subgroups is lower than 50%, we chose the random effect model in this subgroup analysis for consistency. These results showed no evidence of heterogeneity and explained part of the heterogeneity problem of the full analysis. As four studies from the antidiabetic treatment subgroup were located in China [11–14], we next performed pooled analysis, and the result was similar to that for the antidiabetic treatment subgroup [Figure 2(c), MD = −0.27; 95% CI (−0.34 and −0.20), *P* < 0.00001, *I* ^2^ = 0%].

We also evaluated the heterogeneity for other relevant factors across studies (Table 2). Subgroup analysis based on average age (<55 or ≥55), study type (case-control or cross-sectional), and study quality (<7 or ≥7) revealed no statistical significance between the type 2 diabetes group and controls. However, lower nesfatin-1 levels in type 2 diabetes were evident in some subgroups, such as the homeostasis model assessment of insulin resistance (HOMA-IR) ratio (≥4), diagnosis criteria (ADA), and the blood sample source (serum). Further evidence is needed to examine potential differences for these subgroups.

## 4. Discussion

This is the first meta-analysis examining nesfatin-1 levels in diabetes mellitus, and the first meta-analysis of nesfatin-1 using studies obtained from popular databases. Using this analytical method, we identified the cause of the discrepancy in previous studies of circulating nesfatin-1 levels in type 2 diabetes patients.

Li et al. first investigated the fasting plasma levels of nesfatin-1 in type 2 diabetes patients and found that fasting nesfatin-1 levels were significantly lower in the type 2 diabetes group compared with the levels in healthy subjects [12]. Several subsequent studies also reached the same conclusion [10, 11, 13, 14]. However, Zhang et al. reached the opposite conclusion [9]. They found that only newly diagnosed type 2 diabetes patients showed increased circulating nesfatin-1 levels compared to the controls. Guo et al. reported similar results [8]. Two studies that were identified during the literature search, but were not included in the meta-analysis because they used different research markers, both suggested that nesfatin levels were higher in newly diagnosed patients than in controls [23, 24]. Nakata et al. reviewed the conflicting data and claimed that the discrepancy in the conclusions was caused by differences in BMI and insulin resistance of the patient [25]. However, Guo et al. and Zhang et al. both concluded that the discrepancy might be caused by differences in study design, including patient selection and experimental conditions [8, 9]. Khalili et al. agreed with this suspicion in their review in 2016 [26]. Therefore, multiple factors may contribute to the different results.

The overall meta-analysis did not show a significant relationship of circulating nesfatin-1 levels for type 2 diabetes patients, and we suspect that this result was mainly caused by the substantial heterogeneity (*I* ^2^ = 95%). Thus, we operated numerous subgroup analyses to search for the source of heterogeneity. Subgroup analysis was based on several factors that may relate to nesfatin-1 levels, including whether patients received antidiabetic treatment for type 2 diabetes, regional differences, blood sample source, diagnosis criteria, study type, and study quality.

As shown in Figure 2(b), circulating nesfatin-1 levels were significantly lower in type 2 diabetes patients receiving antidiabetic treatment, but newly diagnosed type 2 diabetes patients exhibited considerably higher levels of circulating nesfatin-1. The significantly reduced heterogeneity in both groups suggested that treatment for type 2 diabetes is the main source of heterogeneity.

Nesfatin-1 is reported to exert an antihyperglycemic effect under impaired glucose metabolism conditions [2]. It may also act in the brain to upregulate insulin sensitivity [3] and increase insulin release in beta cells in response to hyperglycemia [4]. Nesfatin-1 was also found to inhibit food intake in the central nervous system [1], but the regulatory mechanism remains unclear. Since nesfatin-1 can cross the brain-blood-barrier [5] and hypothalamic nesfatin-1 can significantly inhibit food intake [1], Li et al. proposed that diabetic polyphagia is caused by decreased circulating nesfatin-1 levels [12]. Studies have also shown that nesfatin-1 can stimulate the lipid metabolism and exhibit anti-inflammatory effects [27]. Since type 2 diabetes often occurs with obesity [6], insulin action dysfunction [7], eating disorders [28], and inflammation [29] and recent studies have reported anti-inflammatory effects of nesfatin-1 [30, 31]; a reasonable model is that there are increased circulating nesfatin-1 levels in patients with newly diagnosed type 2 diabetes, reducing blood glucose, inhibiting food intake, increasing lipid metabolism, and countering inflammation. In patients with type 2 diabetes who are receiving antidiabetic treatment, circulating nesfatin-1 levels are decreased, as antidiabetic treatments target the reduction of blood glucose, increased insulin sensitivity, and controlled food intake.

Although Li et al. reported that fasting plasma levels of nesfatin-1 were positively correlated with age [12], our subgroup analysis for age did not identify reduced heterogeneity. Similarly, the partly high levels of heterogeneity in BMI, HOMA-IR, blood sample source, diagnosis criteria, study type, and study quality subgroups suggested that these factors might not contribute to the observed heterogeneity in the overall meta-analysis. Due to the limited number of included studies, more researches in this area are needed to come to the conclusion.

Since the long duration of type 2 diabetes is accompanied with antidiabetic treatment and the newly diagnosed type 2 diabetes corresponds to a shorter duration, subgroup analysis of the acceptance of antidiabetic treatment can partially illustrate the relationship between circulating nesfatin-1 levels and the duration of the disease. Although circulating nesfatin-1 levels may be affected by the duration of type 2 diabetes, this information was only included in three studies, preventing subgroup or regression analysis.

The present meta-analysis has several limitations. First, as only seven studies were included in this meta-analysis, the potential significance in the overall meta-analysis may not be shown. Second, we tried but failed to get the original data of the included studies, so the accuracy of the data cannot be guaranteed. Third, as most of these studies were performed in China (shown as Figure 2(b)), regional differences may also lead to differences in results and further studies in different regions are required to test this hypothesis. Additionally, only case-control and cross-sectional data were searched from the databases and included here, which makes it difficult to evaluate the causal association between nesfatin-1 levels and the progression of type 2 diabetes. Further examination of this temporality requires more evidence-like cohort studies.

## 5. Conclusion

From this systematic review and meta-analysis, we concluded that there is a relationship between circulating nesfatin-1 levels and type 2 diabetes, and that circulating Nesfatin-1 levels may depend on whether the subjects received antidiabetic treatment or partly relate to the disease duration. Type 2 diabetes patients receiving antidiabetic treatment or with long disease duration exhibited lower circulating nesfatin-1 levels, and early-stage type 2 diabetes was associated with an elevated nesfatin-1 level, possibly due to a compensation mechanism for blood glucose and food intake.

This meta-analysis suggested a potential role of nesfatin-1 in type 2 diabetes, and the nesfatin-1 level may be a good indicator of the progression type 2 diabetes and a target for antidiabetic treatment. Although the roles of nesfatin-1 in the pathogenesis of type 2 diabetes are presently not well understood, nesfatin-1 therapy may be an effective future treatment for obesity and diabetes.

## Figures and Tables

**Table 1:** Characteristics of studies included in the meta-analysis.

Study	Region	Study type	Sample	Methods	T2DM criteria	Antidiabetic treatment	Duration	Sample size (M/F)		Nesfatin-1(*μ*g/l)	
								Case	Control	Case	Control
Algul et al. [10]	Turkey	Cross-sectional	Serum	ELISA	ADA	Yes	—	20 (−/−)	20 (−/−)	0.867 ± 0.09	1.094 ± 0.31
Dai et al. [11]	China	Cross-sectional	Serum	ELISA	ADA	Yes	—	55 (29/26)	48 (23/25)	1.26 ± 0.319	1.51 ± 0.467
Guo et al. [8]	China	Case-control	Plasma	ELISA	WHO	No	—	64 (34/30)	63 (31/32)	2.01 ± 0.64	1.35 ± 0.43
Li et al. [12]	China	Case-control	Plasma	ELISA	ADA	Yes	6.44 ± 0.91	47 (23/24)	20 (13/7)	0.99 ± 0.55	1.48 ± 0.72
Liu et al. [13]	China	Case-control	Plasma	ELISA	WHO	Yes	9.03 ± 3.48	30 (16/14)	30 (14/16)	0.73 ± 0.14	1.00 ± 0.23
Tang et al. [14]	China	Case-control	Serum	ELISA	WHO	Yes	4.56 ± 1.73	38 (18/20)	40 (21/19)	0.86 ± 0.42	1.1 ± 0.34
Zhang et al. [9]	China	Cross-sectional	Plasma	ELISA	WHO	No	—	74 (39/35)	73 (36/37)	1.91 ± 0.79	1.41 ± 0.58

ADA: American Diabetes Association; WHO: World Health Organization; —: not available or not reported. Data are presented as mean ± SD.

**Table 2:** Summary risk estimates of circulating nesfatin-1 levels and type 2 diabetes mellitus.

	Studies	Random effects SMD (95% CI)	*I* ^2^ (%)	P for heterogeneity
Overall	7	−0.16 (−0.57, 0.24)	98	<0.00001
Subgroup analysis				
Age				
<55	3	0.00 (−0.41, 0.42)	94	<0.00001
≥55	4	−0.08 (−0.53, 0.37)	96	<0.00001
BMI				
<25	2	−0.25 (−0.36, 0.13)	0	<0.0001
≥25	5	−0.04 (−0.36, 0.44)	96	<0.00001
HOMA-IR ratio				
<4	3	0.29 (−0.37, 0.95)	98	<0.00001
≥4	2	−0.23 (−0.34, −0.12)	0	0.91
Unknown	2	−0.32 (−0.53, −0.11)	33	0
Blood sample				
Plasma	4	−0.03 (−0.11, 0.05)	0	0.98
Serum	3	−0.24 (−0.33, −0.15)	97	<0.00001
T2D criteria				
WHO	4	0.16 (−0.31, 0.63)	97	<0.00001
ADA	3	−0.26 (−0.36, −0.16)	0	0.39
Study type				
Case-control	4	−0.08 (−0.53, 0.37)	96	<0.00001
Cross-sectional	3	0.00 (−0.41, 0.41)	94	<0.00001
Study quality				
Good	6	−0.01 (−0.35, 0.33)	96	<0.00001
Moderate	1	—	—	—

## Supplementary Materials

Supplementary 1 Table 1: baseline characteristics of the enrolled studies.

Supplementary 2 Table 2A: quality assessment of included case-control studies. Table 2B: quality assessment of included cross-sectional studies.
