# Supplementary material for: Circulating Nesfatin-1 Levels and Type 2 Diabetes: A Systematic Review and Meta-Analysis
Source: J Diabetes Res. 2017 Dec 28;2017:7687098. doi: 10.1155/2017/7687098 (PMC5763168; doi:10.1155/2017/7687098)
Supplement: Supplementary 1 — Table 1: baseline characteristics of the enrolled studies. [file 7687098.f1.docx]

| **Supplementary Table 1** Baseline characteristics of the enrolled studies | | | | | | |  |  |  |  |  |  |
| --- | --- | --- | --- | --- | --- | --- | --- | --- | --- | --- | --- | --- |
| Study | Age (year) | | BMI (kg/m2) | | FBG (mmol/L) | | FINS (mU/L) | | HOMA-IR ratio | | HbA1c (%) | |
|  | Case | Control | Case | Control | Case | Control | Case | Control | Case | Control | Case | Control |
| Algul, 2016 [13] | 40.6±6 .261 | 39.2±5. 367 | 29.6±3 .578 | 24.1±3. 13 | 12.99± 2.73 | 5.05±0. 25 | 10.14±4 .02 | 4.86±3. 58 | 6.694± 2.37 | 1.057±0 .581 | 9.11±1 .431 | 4.87±0. 358 |
|  |  |  |  |  |  |  |  |  |  |  |  |  |
| Dai, 2017 [14] | 57.45± 7.89 | 56.73±7 .50 | 23.67±3.55 | 23.37±2.01 | 7.68±1 .22 | 5.15±0. 40 | — | — | — | — | — | — |
|  |  |  |  |  |  |  |  |  |  |  |  |  |
| Guo, 2013 [11] | 55±11 | 54±12 | 25.8±3.7 | 25.7±2.7 | 11.0±3 .7 | 5.3±0.4 | 8.70±4. 38 | 7.95±3. 45 | 3.81±1.83 | 1.92±0.87 | 8.9±2. 4 | 5.7±0.4 |
|  |  |  |  |  |  |  |  |  |  |  |  |  |
| Li, 2010 [15] | 59.55±12.34 | 47.30±10.24 | 26.46±3.154 | 24.96±4.114 | 8.41±0 .36 | 5.33±0. 26 | 4763.3± 338.4* | 2146.6± 229.7* | — | — | — | — |
|  |  |  |  |  |  |  |  |  |  |  |  |  |
| Liu, 2014 [16] | 62.23±8.11 | 59.13±9.45 | 25.79±2.64 | 23.71±2.65 | 7.37 ± 1.15 | 5.00 ± 0.46 | 7.72 ± 0.86 | 5.56 ± 1.27 | 2.54±0.55 | 1.24±0.33 | 7.23 ± 1.27 | 5.63 ± 0.61 |
|  |  |  |  |  |  |  |  |  |  |  |  |  |
| Tang, 2015 [17] | 51.38±7.56 | 52.61±5.16 | 22.76±4.33 | 22.15±5.64 | 9.68±3 .52 | 4.47±0. 81 | 11.36±4 .58 | 7.21±2. 36 | 6.49±1.18 | 1.58±0.57 | 8.45±2 .16 | 4.58±1. 42 |
|  |  |  |  |  |  |  |  |  |  |  |  |  |
| Zhang, 2012 [12] | 54±11 | 51±7 | 25.0±3.7 | 24.5±3.6 | 10.9 ± 3.8 | 5.4 ± 0.4 | 8.26 ± 4.25 | 7.74 ± 2.85 | 3.79±1.77 | 1.85±0.66 | 8.8 ± 2.4 | 5.5 ± 0.4 |
|  |  |  |  |  |  |  |  |  |  |  |  |  |
| *BMI* body mass index, *HOMA-IR* homeostasis model assessment of insulin resistance, *HbA1c* glycosylated hemoglobin, *FBG* fasting blood glucose, *FINS* fasting plasma insulin. Data are presented as mean±SD. *(pg/ml) | | | | | | | | | | | | |
