# Supplementary material for: Circulating Nesfatin-1 Levels and Type 2 Diabetes: A Systematic Review and Meta-Analysis
Source: J Diabetes Res. 2017 Dec 28;2017:7687098. doi: 10.1155/2017/7687098 (PMC5763168; doi:10.1155/2017/7687098)
Supplement: Supplementary 2 — Table 2A: quality assessment of included case-control studies. Table 2B: quality assessment of included cross-sectional studies. [file 7687098.f2.docx]

| **Supplementary Table 2A** Quality Assessment of Included Case-control Studies | | | | | | | | | | | | | | | | | | | | | |  |
| --- | --- | --- | --- | --- | --- | --- | --- | --- | --- | --- | --- | --- | --- | --- | --- | --- | --- | --- | --- | --- | --- | --- |
| Study | | A | | B | | C | | D | | E | | F | | G | | H | | Total quality score | | | |  |
| Guo 2013  [8] | | 1 | | 0 | | 0 | | 1 | | 2 | | 1 | | 1 | | 1 | | 7 | | | |  |
| Li 2010  [12] | | 1 | | 1 | | 0 | | 1 | | 1 | | 1 | | 1 | | 1 | | 7 | | | |  |
| Liu 2014  [13] | | 1 | | 1 | | 0 | | 1 | | 1 | | 1 | | 1 | | 1 | | 7 | | | |  |
| Tang 2015  [14] | | 1 | | 1 | | 0 | | 1 | | 2 | | 1 | | 1 | | 1 | | 8 | | | |  |
| *A:* case defined with independent validation, *B:* representativeness of the cases, *C:* selection of controls from community, *D:* statement that controls have no history of outcome, *E:* cases and controls matched and/or adjusted by factors, *F:* ascertain exposure by blinded structured interview, *G:* same method of ascertainment for cases and controls, *H:* same response rate for both groups.  **Supplementary Table 2B** Quality Assessment of Included Cross-sectional Studies | | | | | | | | | | | | | | | | | | | | | |  |
|  |  |  |  |  |  |  |  |  |  |  |  |  |  |  |  |  |  |  |  |  |  |  |
|  |  |  |  |  |  |  |  |  |  |  |  |  |  |  |  |  |  |  |  |  |  |  |
|  |  |  |  |  |  |  |  |  |  |  |  |  |  |  |  |  |  |  |  |  |  |  |
| Study | A | | B | | C | | D | | E | | F | | G | | H | | I | | J | K | Total quality score | |
| Algul 2016  [10] | 1 | | 1 | | 0 | | 1 | | 1 | | 1 | | 1 | | 1 | | 1 | | 1 | 0 | 9 | |
| Dai 2016  [11] | 1 | | 1 | | 0 | | 1 | | 1 | | 1 | | 1 | | 1 | | 1 | | 1 | 0 | 9 | |
| Zhang 2012  [9] | 1 | | 1 | | 0 | | 1 | | 1 | | 1 | | 1 | | 1 | | 0 | | 1 | 0 | 8 | |
| *A:* Define the source of information (survey, record review), *B*: List inclusion and exclusion criteria for exposed and unexposed subjects (cases and controls) or refer to previous publications, *C:* Indicate time period used for identifying patients, *D:* Indicate whether or not subjects were consecutive if not population-based, *E:* Indicate if evaluators of subjective components of study were masked to other aspects of the status of the participants, *F:* Describe any assessments undertaken for quality assurance purposes (e.g., test/retest of primary outcome measurements), *G:* Explain any patient exclusions from analysis, *H:* Describe how confounding was assessed and/or controlled, *I:* If applicable, explain how missing data were handled in the analysis, *J:* Summarize patient response rates and completeness of data collection, *K:* Clarify what follow-up, if any, was expected and the percentage of patients for which incomplete data or follow-up was obtained. | | | | | | | | | | | | | | | | | | | | | | |
|  |  |  |  |  |  |  |  |  |  |  |  |  |  |  |  |  |  |  |  |  |  |  |
|  |  |  |  |  |  |  |  |  |  |  |  |  |  |  |  |  |  |  |  |  |  |  |
|  |  |  |  |  |  |  |  |  |  |  |  |  |  |  |  |  |  |  |  |  |  |  |
|  |  |  |  |  |  |  |  |  |  |  |  |  |  |  |  |  |  |  |  |  |  |  |
